# Vertebral Augmentation for Painful Type 4 Osteoporotic Compression Fractures: A Comparative Study

**DOI:** 10.1155/2023/1562892

**Published:** 2023-01-16

**Authors:** Mohamed Abdelrahman Abdalla, Ricardo Rodrigues, Christian Ulbricht

**Affiliations:** ^1^St George's University Hospital, London, UK; ^2^Imperial College Healthcare NHS Trust, London, UK

## Abstract

**Background:**

Type 4 osteoporotic fracture (OF4), according to the classification system of the Spine Section of the German Society for Orthopaedics and Trauma (DGOU), is unstable and requires fixation as per the guidelines of the same group. We evaluated the use of stand-alone vertebral body augmentation (VBA) in pain control of OF4.

**Methods:**

This is a single-centre, in two hospitals, comparative study to evaluate the effectiveness of percutaneous vertebroplasty (PVP) and kyphoplasty (KP) in pain control of OF4. OF4 patients treated with VBA were compared to a conservatively treated control group. The two groups of OF4 were then compared to similar cohort of OF2 and OF3 patients who were treated by either VBA or expectantly.

**Results:**

A total of 78 cases were studied. VBA of OF4 showed a statistically significant better pain control than conservative treatment. The response of this group of fractures to VBA was similar to that of OF2 and 3.

**Conclusion:**

VBA can provide satisfactory pain control for OF4 patients.

## 1. Introduction

The management of osteoporotic vertebral compression fractures (VCFs) remains a challenge and a controversy among healthcare providers. Most of these fractures do not necessarily require surgical decompression or fixation and can be managed conservatively. Nevertheless, conservative management in this group of patients, often elderly with other comorbidities, can result in several complications. These include complications of bed rest, respiratory compromise, spinal deformity [1], and the risks of opioid use [2].

VBA, although still of controversial value in VCFs, can provide rapid pain relief. The hypothesized mechanisms are fracture stabilization and chemical and thermal damage of the sensory nerve fibres by the injected cement [3].

The Spine Section of the German Society for Orthopaedics and Trauma (DGOU) classifies these fractures into five types [4] This classification system treats osteoporotic fractures as a separate entity from other thoracolumbar fractures. It has helped establish the DGOU guidelines for the treatment of each type [5].

As these guidelines state, there are different considerations before choosing the optimum treatment for each case. However, OF3 to OF5 are usually treated surgically. While the guidelines suggested VBA as an option for the treatment of OF3, surgical fixation is indicated for OF4 and OF5 with or without added cement augmentation [5].

We assessed the efficiency and safety of vertebroplasty (VP) and kyphoplasty (KP) for pain control of OF4 in the Spine Unit at both Charing Cross Hospital and St Mary's Hospital. We compared the outcome of VBA for these fractures to conservative management and also to the response of other fracture types to both treatment modalities.

## 2. Methods

We reviewed the patients treated for osteoporotic vertebral compression fractures at the Imperial College Healthcare NHS Trust in the period between March 2016 and February 2020. They were grouped into conservatively treated and VBA patients.

### 2.1. Inclusion and Exclusion Criteria

Inclusion criteria were age above 50, absence of any neurological deficit, radiologically confirmed osteoporosis, and complete preoperative and postoperative medical records. Exclusion criteria were patients who had any other coincident fractures such as hip or lower limb fractures; follow-up carried out at different hospitals; VBA patients who received any other concomitant spinal interventions apart from biopsy, including spinal injections or fixation surgery; patients with other spinal pathology proven from the biopsies, e.g., metastases or haematological malignancies; and incomplete medical records.

Augmentation was performed by either neuro-spine or ortho-spine surgeons or interventional radiologists, under either biplanar X-ray or CT guidance. The bone cement used in all cases was polymethylmethacrylate (PMMA).

Fractures were classified according to the classification system of the Spine Section of the German Society for Orthopaedics and Trauma (DGOU). Hence, the radiology and the type of fracture of each vertebra was assessed separately by two spine surgeons.

All of the augmented cases had a period of failed initial conservative treatment and severe pain (visual analogue score, VAS >7) before the VBA. The duration of this treatment varied between the cases.

### 2.2. Outcome Measures

The outcome of this study was pain score at 6 months from the onset of fracture or from the VBA. Visual analogue scale was used, and the outcome was categorized into either successful (reduction of the VAS by ≥4) or failed (persistent pain or less than 4 points reduction in the score).

## 3. Statistics

One-way ANOVA was used to compare the distribution of the level of fractures between both groups, as well as the type of fracture.

Fischer's exact test was used in order to accommodate the small number of patients in each subgroup. Nevertheless, subgroup analysis of the three types of OF4 could not be performed because of the small number of cases. The same reason excluded OF1 and 5 from the comparative analysis.

Statistics were conducted using Microsoft Excel®. Statistical significance was accepted at *p* < 0.05 level.

## 4. Results

A total of 84 patients, who were treated and followed up only at the Imperial College Healthcare trust hospital, were assessed for eligibility, of whom 78 were included in the study. Three patients were excluded due to insufficient data in their clinical records. Three other patients who had biopsies during VBA that showed metastases were also excluded. The vast majority of VCF cases were referred from and then treated at other hospitals. Their medical records are thus incomplete at Imperial College Hospital and were not assessed in this study.

There was no significant difference in the demographic or clinical characteristics between patients included in and those excluded from the study. Of the patients included in the study, 37 underwent augmentation procedure (vertebroplasty or kyphoplasty) and 41 were treated conservatively. The mean age at VBA was 69.7+/−9.6 years (range, 52–85 years), whilst the mean age in the conservative treatment group was 76.6+/−10.3 years (range, 53–95 years) (*p* > 0.05).

### 4.1. Levels and Types of Fractures

Fractures occurred most frequently in the lower thoracic and upper lumbar levels, L1 being the most common level in both groups (Table 1). 14 patients had multiple levels of VCFs. The level of fracture had no statistical difference between the two groups (one-way ANOVA, *p*=0.37). In regard to the fracture type, OF2 was the most common type in both the conservative (33.3%, *n* = 16) and the VP/KP group (40.2%, *n* = 31) (Table 1). OF4 was the second most common fracture type in the conservative group (31.2%, *n* = 15), whilst the second most common type of fracture in the VP/KP group was OF3 (33.8%, *n* + 26). However, the distribution of the type of fractures was statistically similar in both groups (one-way ANOVA, *p*=0.40).

Vertebroplasty accounted for 67.6% (*n* = 25) of the patients submitted to surgery, whilst kyphoplasty aggregated 32% of patients (*n* = 12). Complications developed in 40.5% (*n* = 15) of the cases, mostly due to cement leakage (Table 2). In spite of this, no patient suffered any serious postoperative event.

### 4.2. Treatment of OF4 Fractures

In our cohort, a total of 25 patients had OF4 type fractures. Ten patients received VBA (70% vertebroplasty and 30% kyphoplasty) into 15 fractured vertebrae. 70% of them had single level fracture. The other 15 patients were treated conservatively (86.7% for single level fracture). The average age of OF4 group patients was 78.4 (+/−11.8) years in the conservative group and 74.7 (+/=6.7) years in the VP/KP group (Table 3). Sex distribution in both conservative and VBA groups was nearly similar (53.3% and 50% females, respectively).

OF4 showed poor pain alleviation with conservative treatment (only 1 out of the 15 patients; 6.7%), but statistically significant improvement with VBA (90%, *p* < 0.01). The rate of complications of VBA in the OF4 cohort was 40%. All of these complicated cases had cement leakage into the disc space and/or surrounding tissues including paravertebral veins but with no untoward effect.

OF4 response to VBA was no different from that of OF2 or 3. The three groups showed similar response to VBA with no statistically significant difference (*p*=0.49 and *p*=0.23, respectively) (Table 4).

## 5. Discussion

“There have been few more debated topics in medicine over the last decade than percutaneous vertebroplasty” [6].

Osteoporotic VCFs are common in elderly and are more prevalent among postmenopausal women as found in this study. The most commonly fractured area is the thoracolumbar junction, as shown in a recent report [7]. T12 and L1 harboured 35.06% of the fractures in our series.

There have been few classification systems that recognized this group of fractures as a separate entity [8, 9] [10]. Spine Section of the German Society for Orthopaedics and Trauma classified them into five types [4]. The same group has also published treatment guidelines based on this classification [5]. As a general rule, OF4 and 5 are surgical fractures while OF1 and 2 are conservatively treatable and OF3 can be treated either way. Surgical treatment in this context means screw fixation with or without cement augmentation [5]. As this system is relatively new, there have been few studies on the treatment of OF4. Short segment fixation augmented by cement [11] showed good results comparable to the previously studied vertebroplasty in combination with intermediate bilateral pedicle screw fixation [12].

In our studied group of patients, none had surgical fixation.

We evaluated the use of stand-alone VBA for pain control of OF4 and compared it to the conservative treatment as well as to the response of the other fracture types (OF2 and 3) to VBA. There was no case in the studied OF4 that was surgically fixed. OF5 cases were few and no meaningful data could be retrieved from their analysis.

The most common practice in our unit has been initial period of expectant management before offering VBA. However, nine patients were offered intervention straight away when seen in the clinic because of suspicion of malignancy. In theory, some of these cases could have improved with conservative treatment had it been adopted. However, they all had severe pain on presentation and therefore were included to assess their response to VBA.

On the other hand, our results showed a good outcome to VBA even with delayed treatment which contradicts the recommendations of VAPOUR (safety and efficacy of vertebroplasty for acute painful osteoporotic fractures) study [13].

VBA also showed statistically significant difference over conservative treatment in pain control in the three studied fracture types. This response was no different in OF4 than that in OF2 and 3. Because of the small number of OF4 cases in this series, subgroup analysis to examine the response of each subtype to cement augmentation could not be performed. The same reason did not allow comparison between PVP and KP for OF4.

Overall, we found excellent outcome of both PVP and KP in pain control of OF4. The response did not differ either with age or sex of the patients nor was it affected by the level of the treated vertebral body, whether single or multiple levels were injected or if the procedure was performed by a surgeon or interventionist.

This study had some limitations. It is retrospective with relatively small number of cases. Further studies are needed to prospectively evaluate the use of VBA only for the pain control of OF4 including its three subtypes.

## 6. Conclusion

OF4, although classified as unstable fractures, can still be treated with VBA and achieve satisfactory pain control. Early treatment of these fractures is recommended because of the high likely failure of the conservative management.

## Figures and Tables

**Table 1:** Types and levels of fracture in the conservative and VP or KP groups.

	Conservative (*n* of VCF = 48)	VP or KP (*n* of VCF = 75)
Type of fracture, % (*n*)		
OF1	6.3% (3)	0% (0)
OF2	33.3% (16)	41.3% (31)
OF3	22.9% (11)	34.7% (26)
OF4	31.2% (15)	20% (15)
OF5	6.3% (3)	4% (3)
Level of fracture, % (*n*)		
T3	2.1% (1)	0% (0)
T5	4.2% (2)	2.7% (2)
T6	2.1% (1)	2.7% (2)
T7	2.1% (1)	4% (3)
T8	0% (0)	2.7% (2)
T9	4.2% (2)	4% (3)
T10	4.2% (2)	1.3% (1)
T11	4.2% (2)	4% (3)
T12	16.7% (8)	13.3% (10)
L1	20.8% (10)	18.7% (14)
L2	16.7% (8)	16% (12)
L3	8.3% (4)	16% (12)
L4	6.2% (3)	10.6% (8)
L5	8.3% (4)	4% (3)
Multilevel fracture, % (*n*)		
Yes	17.1% (7)	45.9% (17)
No	82.9% (34)	54.1% (20)

**Table 2:** Technique and complications of VBA.

	VP or KP (*n* = 37)
Type of procedure, % (*n*)	
Vertebroplasty	67.6% (25)
Kyphoplasty	32.4% (12)
Injection laterality, % (*n*)	
Unilateral	72% (54)
Bilateral	22.7% (17)
N/A	5.3% (4)
Amount of cement, mL (±SD)	3.6 (±1.7)
Complications, % (*n*)	
None	59.5% (22)
Intradiscal cement leakage	21.6% (8)
Paravertebral veins cement leakage	8.1% (3)
Surrounding tissues cement leakage	5.4% (2)
Late adjacent-level fractures	5.4% (2)

**Table 3:** Demographics and level of OF4 type fractures in conservative and VBA groups.

	Conservative (*n* = 15)	VP or KP (*n* = 10)
Age (years), average (SD)	78.4 (±11.8)	74.7 (±6.7)
Sex: female, % (*n*)	53.3% (8)	50% (5)
Level of fracture, % (*n*)		
T5	0% (0)	6.7% (1)
T6	13.3% (2)	6.7% (1)
T7	6.7% (1)	6.7% (1)
T8	0% (0)	6.7% (1)
T9	6.7% (1)	6.7% (1)
T11	0% (0)	6.7% (1)
T12	13.3% (2)	26.7% (4)
L1	13.3% (2)	20% (3)
L2	26.7% (4)	0% (0)
L3	13.3% (2)	6.7% (1)
L4	0% (0)	6.7% (1)
L5	6.7% (1)	0% (0)
Yes	13.3% (2)	30% (3)
No	86.7% (13)	70% (7)

**Table 4:** Response to vertebral body augmentation by type of osteoporotic vertebral fracture.

	OF2	OF3	OF4
Management			
Successful	13	14	9
Failed	3	6	1
*p* ^*∗*^	0.49	0.23	—

^*∗*^Compared to OF4-type fractures.

## Data Availability

The data used in this study are available from the corresponding author upon request.
